# Supplementary material for: Single-Shot 3D Topography of Transmissive and Reflective Samples with a Dual-Mode Telecentric-Based Digital Holographic Microscope
Source: Sensors (Basel). 2022 May 17;22(10):3793. doi: 10.3390/s22103793 (PMC9144696; doi:10.3390/s22103793)

Single-shot 3D topography of transmissive and reflective samples with a dual-mode telecentric-based digital holographic microscope: supplementary material

ANA DOBLAS,[1,*] CHARITY HAYES-ROUNDS,[1] ROHAN ISAAC,[2] AND FELIO PEREZ[3]

[1]*Department of Electrical and Computer Engineering, The University of Memphis, Memphis, TN 38152, USA*
[2]*FedEx Institute of Technology, The University of Memphis, Memphis, TN 38152, USA*
[2]*Material Science Lab, Integrated Microscopy Center, The University of Memphis, Memphis, TN 38152, USA*
**adoblas@memphis.edu*

Abstract: This document provides supplementary information to "Single-shot 3D topography of transmissive and reflective samples with a dual-mode telecentric-based digital holographic microscope". Here, we include the list of optical and optomechanical components from Thorlabs, one of the major vendors, and the optical schematics for each alignment step.

1. Optical and Optomechanical Components

To ease the implementation of the dual-model common-path DHM system using a Fresnel biprism, Table S1 is the list of the components required to construct the system using products from Thorlabs, which is one of the major vendors of optical elements and optomechanical components. In the list, the camera and Fresnel biprism are purchased through Edmund Optics and Newlight Photonics, respectively.

Table S1. List of hardware to implement the dual-mode common-path DHM system.

Unit	Component	Part number	Manufacturer
3	Construction Rail, 500 mm length	XT66-500	Thorlabs
6	XT66 Horizontal Mounting plate	XT66P3	Thorlabs
2	Collimated laser-diode-pumped DPSS laser module with power supply, 532 nm, up to 4.5mW	CPS532	Thorlabs
2	Laser diode mount	KAD11F	Thorlabs
9	SM1-Threaded 30 mm Cage Plate for 66 mm Rails	RCA1	Thorlabs
2	Snap-On 30 mm Cage Mounting Bracket for 66 mm Rails	RCA2	Thorlabs
1	Adapter with External M25.5×0.5 Threads and Internal SM1 Threads for Nikon microscope objective lenses	SM1A25	Thorlabs
1	40× Nikon Plan Fluorite Imaging Objective, 0.75 NA, 0.66 mm WD	N40X-PF	Thorlabs
1	f=200 mm, Ø1" Achromatic Doublet, ARC: 400-700 nm	AC254-200-A-ML	Thorlabs
2	f=75mm, Ø1" Achromatic Doublet, SM1-Threaded Mount, ARC: 400-700 nm	AC254-075-A-ML	Thorlabs
1	Ø1" Mounted Pinhole, 30 ± 2 µm Pinhole Diameter, Stainless Steel	P30K	Thorlabs
1	XY Translator with Micrometer Drives, Metric, for aligning the lateral position of the pinhole	ST1XY-S/M	Thorlabs
1	Ace-IMX 183, 1inch, C-Mount, 5472x3648 px^2, 2.4μm^2 pixel size, 17fps, Mono, CMOS, USB 3.0, Rolling Shutter	11-502	Edmund Optics
1	Adapter with External C-Mount Threads and External SM1 Threads for mounting the camera	SM1A39	Thorlabs

1	BK7 Fresnel prism 20x20x1 mm apex angle 170 deg	FBP2020G-170	Newlight Photonics
1	25×36 mm 50:50 UVFS Plate Beamsplitter, Coating: 400 - 700 nm, t = 1 mm	BSW10R	Thorlabs
1	30 mm Cage Cube with Filter Mount (Metric)	CM1-DCH/M	Thorlabs
1	48.6 mm×48.6 mm Kinematic Platform Mount for mounting the plate beamsplitter	KM100B/M	Thorlabs
1	Small Adjustable Clamping Arm, M4 x 0.7 Threaded Post	PM3/M	Thorlabs
1	Extension Post for PM3/M Clamping Arm, M4 x 0.7 Threaded	PM3SP/M	Thorlabs
1	Universal Post Holder Adapter	UPHA	Thorlabs
1	Ø12.7 mm Aluminum Post, M4 Setscrew, M6 Tap, L = 150 mm	TRA150/M	Thorlabs
1	10× Beam Expander, 400-650nm	GBE10-A	Thorlabs
1	Adapter with External SM2 Threads and Internal M43 x 0.5 Threads	SM2A30	Thorlabs
1	60 mm Cage Plate, SM2 Threads, 0.5" Thick, M4 Tap (Two SM2RR Retaining Rings Included)	LCP01/M	Thorlabs
2	Cage Assembly Rod, 1" Long, Ø6 mm for mounting the beam expander	ER1	Thorlabs
1	Cage Assembly Rod, 6" Long, Ø6 mm, 4 Pack	ER6-P4	Thorlabs
1	Cage Assembly Rod, 3" Long, Ø6 mm, 4 Pack	ER3-P4	Thorlabs
1	Cage Assembly Rod, 1.5" Long, Ø6 mm, 4 Pack (auxiliary for building the cage)	ER1.5-P4	Thorlabs
1	XYZ Translation Stage, 50 mm Travel, Metric – *sample stage*	LT3/M	Thorlabs
1	Ø12.7 mm Aluminum Post, M4 Setscrew, M6 Tap, L = 100 mm – *sample stage*	TRA100/M	Thorlabs
1	Quick-Release Rectangular Filter Holder – *sample stage*	SFH2	Thorlabs
2	SM1-Threaded 30 mm Cage Plate for 66 mm Rails – *for aligning the system*	RCA1	Thorlabs
2	SM1 Series Alignment Disk – *for aligning the system*	SM1A7	Thorlabs
1	Shear Plate, 2.5-5 mm Beam Diameter – *for setting the lenses in afocal configuration*	SI050P	Thorlabs
1	Shearing Interferometer with a 10-25.4 mm Beam Diameter Shear Plate – *for setting the lenses in afocal configuration*	SI254	Thorlabs

2. Alignment Protocol of the Dual-mode Digital Holographic Microscopy using a Fresnel biprism

For every optical element, the general alignment procedure consists of checking the lateral and vertical alignment as well as the tilt. For this verification, two alignment targets are used: one target is placed just behind the element to be aligned, and the second target is placed as far as possible of the beam path. After inserting the optical element, one must adjust the height of the element to ensure that the beam comes out vertically level on both alignment targets; this means that the beam is centered vertically on the optical element. Next, one must align laterally the optical element by sliding the optical element laterally across the optical axis to align the beam on the first target, the closest one to the element, as well as adjusting the tilt of the optical element to align the beam on the second target, the furthest one to the element. The lateral alignment is an iterative process as changing the lateral position, and the tilt of the element affect the lateral position of the beam in the two targets. Once the beam is laterally centered on both targets, the optical element will be horizontally and vertically centered and orthogonal to

the optical axis (i.e., no tilt). One can alternatively use irises instead of alignment targets. As one inserts new optical elements (e.g., mirrors and lenses), two irises should be placed. To build the system, the following steps are needed:

Step 0: Mount the optical rails of the transmission-based illumination path onto an optical table. Leave around xxx mm between both rails.

Step 1 – Illumination source of the transmission-based DHM system. Insert a laser source. The laser source should be mounted in a kinematic mount that allows the control of the tilt. Set the two alignment targets along the optical rails to align the laser; one should be closer to the laser head, whereas the other should be placed at the end of the second construction rail. Ensure that the collimated beam emerging of the laser is straight and parallel to the optical axis defined by these two alignment targets.

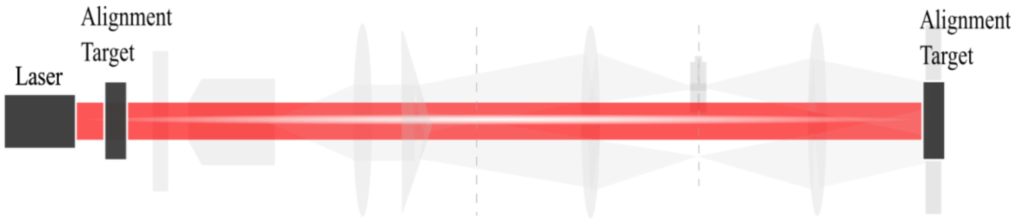

Step 2 – L2 lens from the 4f system. Insert the lens L2 of focal length 75 mm. Align the transverse position of the lens using two alignment targets. Insert the L2 lens approximately 920 mm from the beginning of the construction rail.

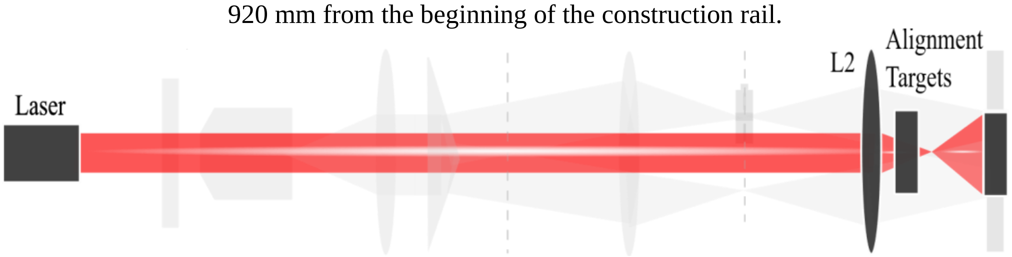

Step 3 – **Camera**. Insert the camera after the L2 lens. Although the camera position is not critical, to reduce the computational processes, the use of DHM systems operating at the image plane is recommended. This means that the camera must be placed at the back focal plane of the L2 lens. To find this position, use the camera's software. It is important to significantly reduce the intensity of the object beam to avoid pixel saturation on the camera, for instance, by inserting a neutral density filter in the optical path. The axial position of the camera can then be set by finding the narrower focus spot generated by the L2 lens. After this position is found, the DHM system operates in the image-plane regime.

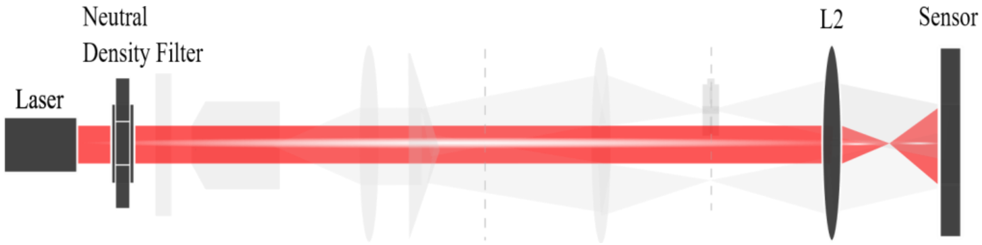

Step 4 – L1 lens from the 4f system. Insert the lens L1 lens of focal length 75 mm. Align the transverse coordinates of the lens using the alignment targets. The lenses L1 and L2 must form an afocal system. Thus, the distance between them is the sum of their focal lengths. Align the

axial position of the L1 so that the beam emerging from the L2 lens is a collimated plane wave. This means that the back focal plane (i.e., image focal plane) of L1 lens coincides with the front focal plane (i.e., object focal plane) of the L2 lens. Insert the shearing interferometer (SI050P) after the L2 lens, and axially displace the L1 lens until straight, parallel lines are observed in the shearing interferometer. Once the axial position of the L1 lens is determined, insert the alignment target after the L1 and verify its lateral alignment. Consider the re-alignment of the L1 lens if the beam is not centered on the alignment target. Connect the components L1, L2 and sensor using rods.

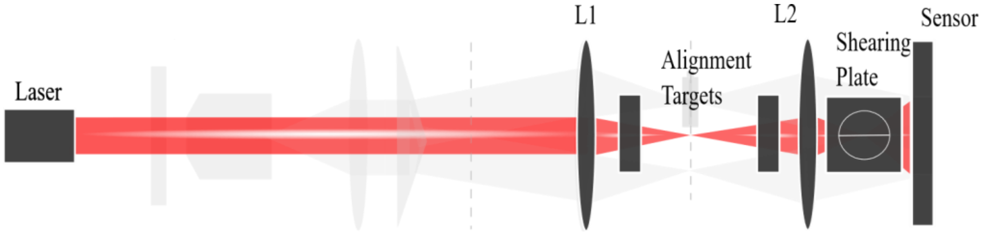

Step 5 – TL lens: Insert the TL lens of focal length 200 mm. Insert the TL lens as close as possible of the end of the first construction rail. Align the transverse coordinates of the lens using the alignment targets. The lenses TL and L1 must form an afocal system. Thus, the back focal plane of TL lens coincides with the front focal plane of the L1 lens. Unfortunately, we cannot use the shearing interferometer after the L1 lens because the beam size is too small. The beam diameter after L1 lens is approximately equal to 3.5mm/($M_{TL\text{-}L1}$) = 1.3125 mm being $M_{TL\text{-}L1} = f_{L1}/f_{TL}$ = 75/200 = 0.375. Therefore, an alternative approach is the insertion of a mirror such that the reflected beam is projected to the furthest wall. Then, we axially displace the TL lens until the smallest focus spot is observed on the wall. Once the axial position of the TL lens is determined, insert the alignment target after the TL and L1 lens to verify the lateral alignment of TL lens.

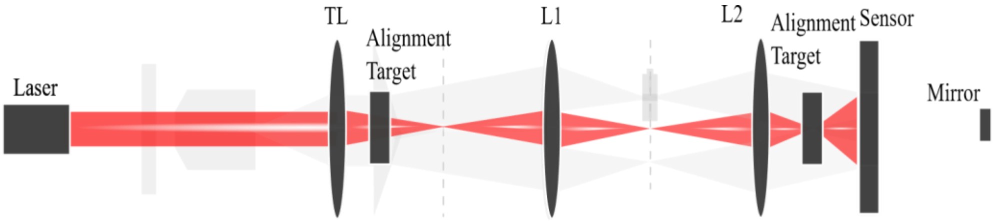

Step 6 – MO lens. Insert an infinity-corrected MO lens before the TL lens. To provide a linear shift-invariant DHM system, the MO and TL must form a telecentric system. Thus, the distance between them is the sum of their focal lengths. Note that the focal length of the MO can be estimated by $f_{MO} = f_{TLM}/M$ where M is the lateral magnification displayed in the MO, and f_{TLM} is the focal length of the TL recommended by the manufacturer. For a Nikon MO lens, f_{TLM} = 200 mm. Use the alignment target to align the MO lens. Align the axial position of the MO so that the beam emerging from the TL is a collimated plane wave. This means that the pupil plane of the MO lens coincides with the front focal plane of the TL lens. Insert a shearing interferometer (SI254) after the TL lens, and axially displace the MO lens until straight, parallel lines are observed in the shearing interferometer. Once the axial position of the MO lens is determined, insert the alignment target after the MO and verify its lateral alignment.

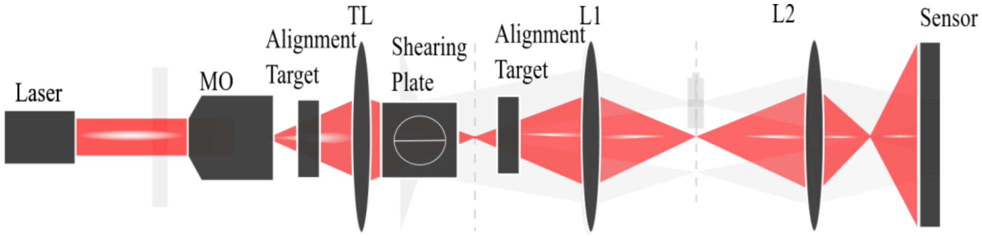

Step 7 – Fresnel biprism. Insert the Fresnel biprism between the TL and L1 lenses. The Fresnel biprism is inserted within a 3D-printed mount which is screwed into a RCA1 mount. The AUTOCAD file for your personal use can be download here: https://www.dropbox.com/home/Students/CHayes-Rounds/Master/Dissertation/Figures. Find the axial position of the Fresnel biprism by observing the fringes' field of view using the camera's software. Displace axially the Fresnel biprism until the fringes' field of view is maximum. The maximum fringes' field of view should be equal to L/2 being L the lateral extension of the Fresnel biprism. If L = 20 mm, the maximum fringes' FOV should be around 10 mm. Fix the position of the Fresnel biprism.

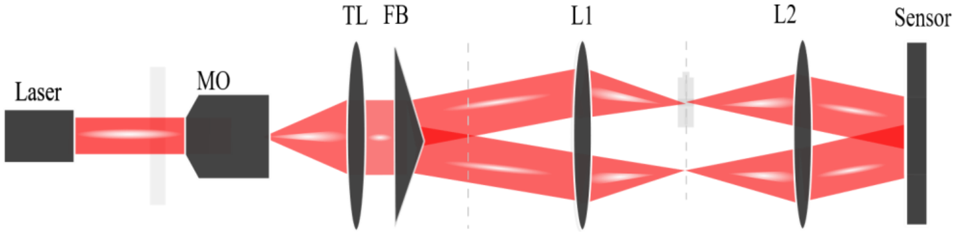

Step 8 – Off-axis configuration. Although the interference angle between both plane waves emerging from the Fresnel biprism is fixed (i.e., period of the fringes cannot be tuned), we need to ensure that the biprism-based DHM system operates in an off-axis regime. The DHM system operates in an off-axis regime if there is no overlap between the different components of the hologram spectrum. The Fourier spectrum of the hologram is composed of three terms: DC term and ±1 terms. For telecentric DHM systems, the compact support of these terms is related to the numerical aperture (NA) of the MO lens and the effective magnification of the telecentric MO-TL imaging system, $M_{eff} = f_{TL}/f_{MO}$, which can be expressed in terms of the objective specifications as $M_{eff} = (f_{TL}\ M)/f_{TLM}$. Particularly, the compact support of these terms is $(2NA)/(\lambda M_{eff})$ for the DC term, and $NA/(\lambda M_{eff})$ for the ±1 terms. The DC term is always centered at the Fourier spectrum, while the position of the ±1 terms depends on the interference angle of the object and reference beams. Note that, in off-axis DHM, the better optimization of the finite space bandwidth of the sensor is achieved when the ±1 orders are placed along the diagonal of the camera's space bandwidth, which is at 45 degrees, allowing their optimal allocation with no overlapping. Whenever possible, consider rotate the biprism to set the components in the hologram's spectrum at 45 degrees.

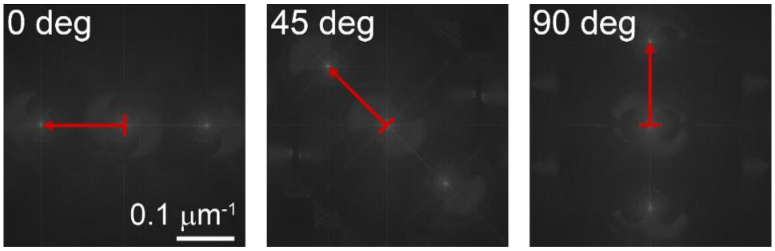

Step 9 – Fine adjustment of the telecentric MO-TL configuration. Knowing that the Fourier transform of a collimated (plane) beam is a Delta function, the telecentric configuration can be verified by observing the Fourier spectrum of the interference between the object and reference waves. Visualizing the center of the ±1 term, the position of the MO can be finely adjusted. The experimental DHM system operates in the telecentric regime if and only if the center of the ±1 term is a maximum peak. This condition must be verified. If there is more than one maximum peak in the Fourier spectrum, the axial position of the MO must be adjusted. If such adjustment of the MO is needed, its alignment must be verified.

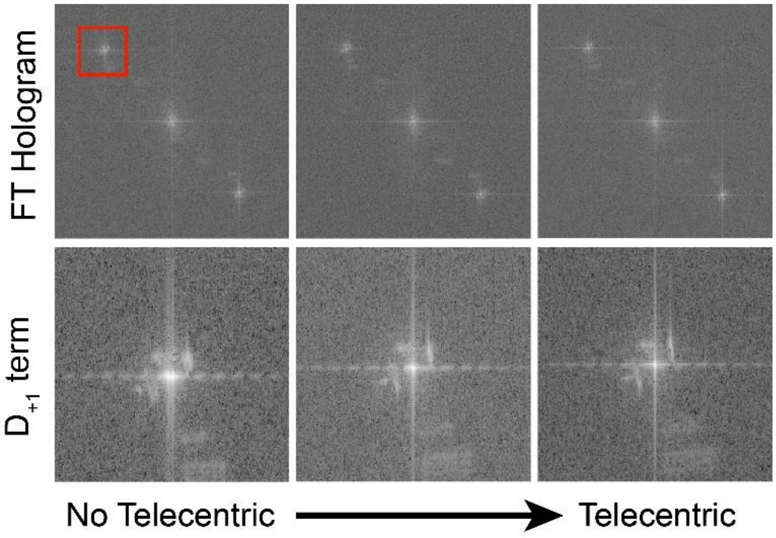

Step 10 – Setting of the spatial filtering system (e.g., pinhole). Remove the rods between the L1-L2-Camera components. Insert the 30-µm pinhole mounted onto a XY translation stage. Drill a manual hole to avoid the spatial filtering of one of the beams emerging from L1 lens (e.g., no filter of the object beam, only the reference beam). Connect the XY translational stage using rods to the RCA2 mounting brackets for 66-mm construction rails. Insert the pinhole between the L1 and the L2 lens as closest as possible to the Fourier plane (e.g., axial plane where the narrowest focus spots are found onto the pinhole mount). Block the object beam. Course lateral alignment of the pinhole by the horizontal and vertical micrometers of the translational stage until the beam emerging the pinhole is laterally uniform (e.g., a uniform plane wave).

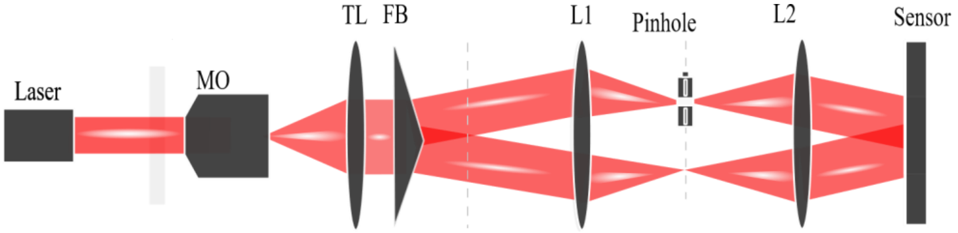

Note that a uniform beam is needed, not a spot of light. If you find a spot, please move the axial position of the pinhole mount. Verify that the lateral alignment of the pinhole. Allow the object beam to interference with the object beam. Make sure that the manual hole is big enough, so the object beam is not clipped. Connect L1-Pinhole-L2-Camera mounts using rods.

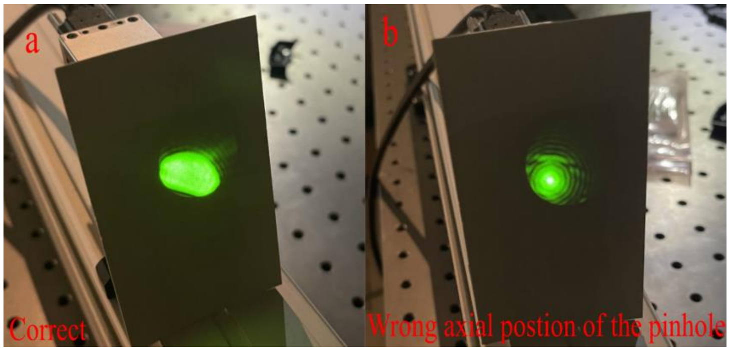

This is the two object replicas with correct spatial filtering. Note that the image of the star information has been filtered out in the reference replica. You can watch these videos to learn more about how to filter a beam: https://www.youtube.com/watch?v=-fmMCMQfXLo and https://www.youtube.com/watch?v=WaAkwVRc7Is.

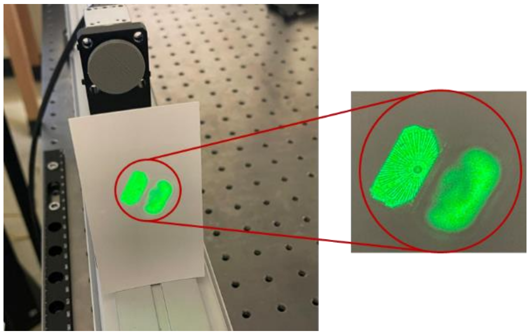

Step 13 – Verification of the performance of the transmission-based DHM system. Insert the sample stage between the laser and the MO lens. After the implementation of the FB-based DHM system, the system's accuracy and resolution limit must be evaluated by imaging a calibrated phase target. For the verification of the imaging capabilities, use of the phase target from Benchmark Technologies is recommended, since it is the first commercially-available quantitative phase target for the evaluation of phase imaging systems. This target has seven distinct feature heights from 50 nm to 350 nm containing familiar microscopy targets such as a focus star with a 400-nm pitch, and a USAF resolution target whose smallest resolvable elements are 274 nm. By reconstructing the phase image of the USAF target, the experimental resolution limit can be estimated as its smallest resolved element. Additionally, the phase image of the USAF target can be used to verify if the DHM system operates at the diffraction limit, comparing the agreement between the experimental and theoretical resolution limits. For coherent imaging systems, the theoretical resolution limit is λ/NA. Alternatively, the experimental resolution limit can also be estimated by imaging a star test from the phase target. Using the star target, the experimental resolution limit is determined by the minimum resolvable star pattern. For this evaluation, since there is not a calibrated table as in the USAF target, the lateral magnification of the imaging system must be determined. The experimental measurement of the lateral magnification can be done by imaging a micrometer (R1L3S2P, Thorlabs). However, one can use the theoretical value of the magnification, $M_{eff} = (f_{TL}\ M)/f_{TLM}$, since the error difference between the experimental and theoretical values, which is usually less than 5%, is negligible.

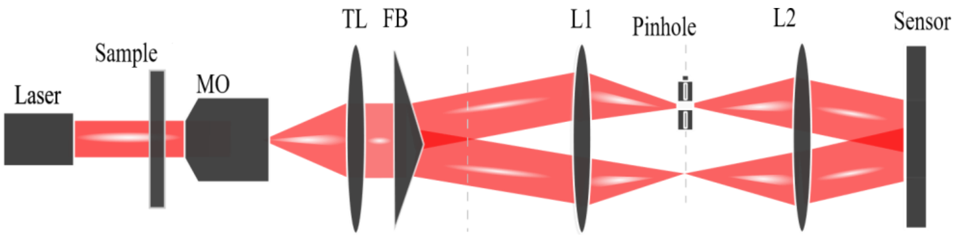

Step 14: Mount the optical rail of the reflection-based illumination path onto an optical table.

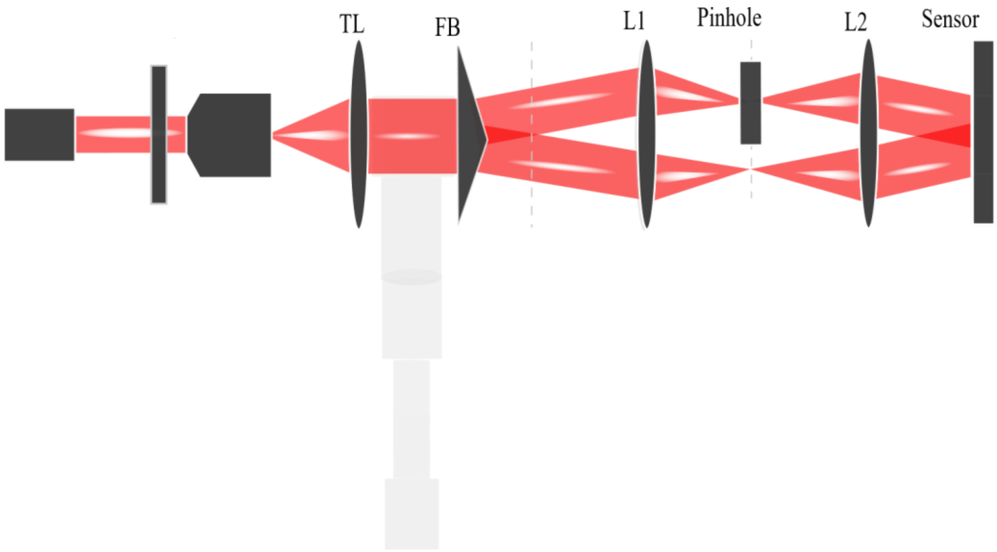

Step 15 – Illumination source of the reflection-based DHM system. Insert a laser source. The laser source should be mounted in a kinematic mount that allows the control of the tilt. Set the two alignment targets along the optical rails to align the laser; one should be closer to the laser head, whereas the other should be placed at the end of the second construction rail. Ensure that the collimated beam emerging of the laser is straight and parallel to the optical axis defined by these two alignment targets.

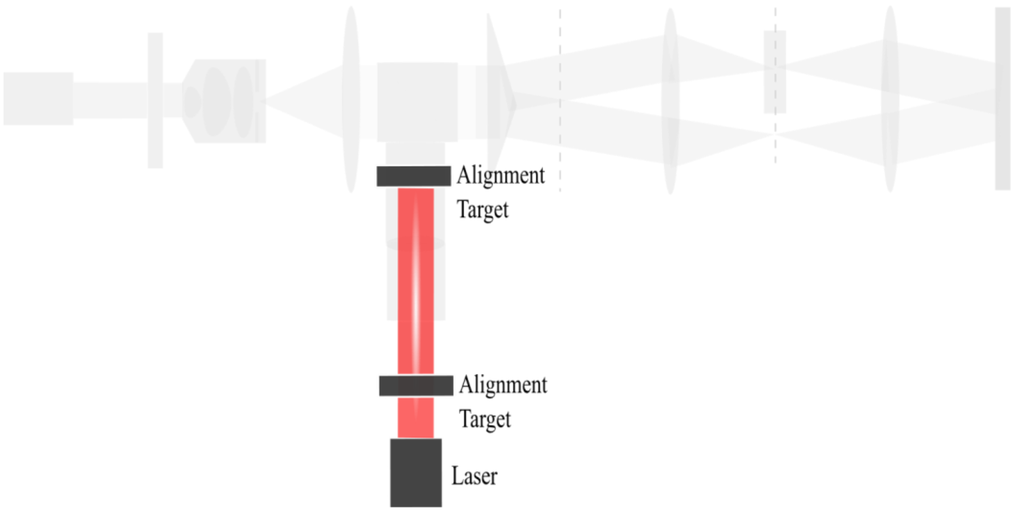

Step 16- Reflection of the illumination beam. Insert the plate beamsplitter (BS) between the TL and FB. Ensure that the reflected beam is going through the TL and MO imaging system. Course adjustment of the beamsplitter ensuring that the reflected beam is not clipped by adjusting the height and horizontal position of the beamsplitter. Set an alignment target closer to the beamsplitter. The second alignment target should be located as closer as possible to the transmission-based illumination source. The beamsplitter must be set at a 45 degrees angle. Change the height of the beamsplitter, ensuring that the illumination beam from the reflection path impacts roughly in the center of it. Set beamsplitter's tilt so that the reflected beam is centered in the furthest alignment target. Now align the lateral position of the beamsplitter centering the beam in the closest alignment target. Slide the beamsplitter to center horizontally the beam on the first target. Then, change the horizontal tilt of the beamsplitter to set the beam in the second target. Repeat this procedure until the reflected beam is aligned in both irises.

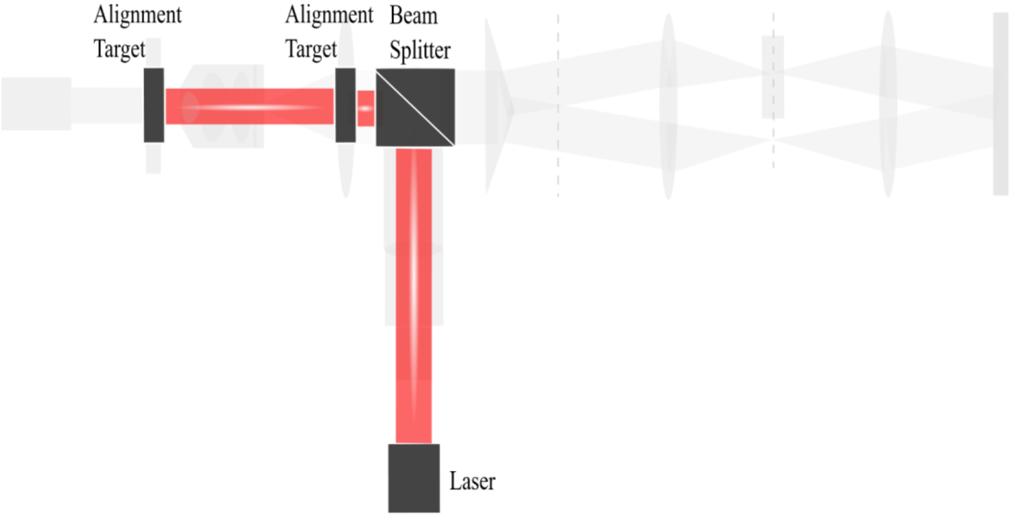

Step 16 – Increase the reflected beam diameter. Insert the 10× beam expander (BE) between the laser and beamsplitter. To align the beam, insert the alignment targets after the beam expander (same positions as in step 15). Adjust the beam expander until the beam is centered.

Step 17 – Fine tune of the collimation in the beam expander. Insert a plane mirror as the object. Adjust the sliding lens so that the beam reflected from the plane mirror and passing through the MO and TL lenses is collimated. Insert the shear plate after the TL lens (right before the Fresnel biprism) and ensure that we observe straight, parallel fringes. In the event that this is not possible, insert of an additional mirror such that the emerging beam from the MO lens is projected to the furthest wall. Then, we axially displace the sliding lens until the smallest focus spot is observed on the wall.

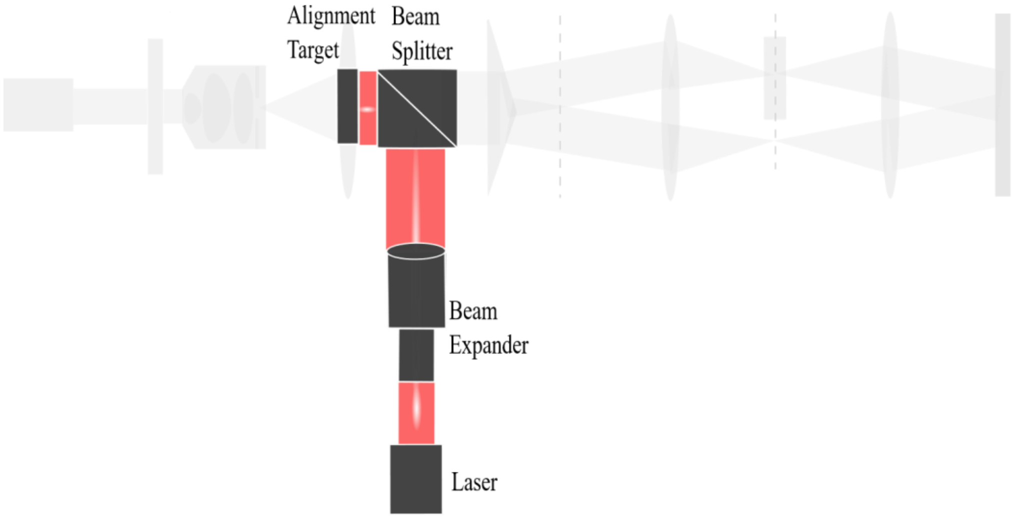

Step 18 – Verification of the performance of the reflection-based DHM system. Insert a reflective sample at the working distance of the MO lens. Verify that the 30-µm pinhole mounted is aligned to the reflection-based imaging modality. If it is not, fine tune the position of the beamsplitter or the beam expander. Another alternative is aligning the pinhole for the reflection-based imaging modality. But, be aware that every time that you changes the imaging modality, there is need to adjust the lateral position of the pinhole. For the verification of the imaging capabilities, use of the high-resolution USAF target in which you can add a 40-nm reflective layer. Read the manuscript for more details.

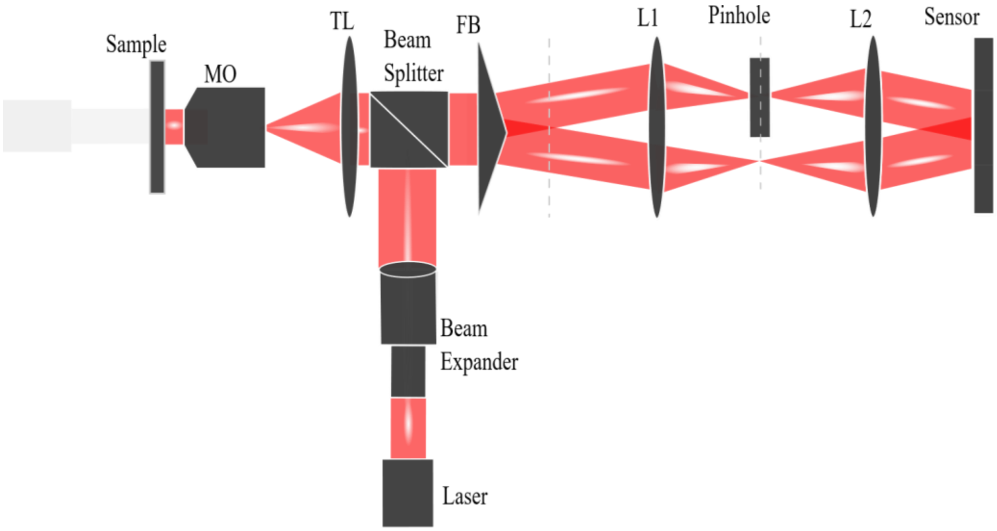

Supplement: Supplementary file 1 [file sensors-22-03793-s001.zip › sensors-1702012-supplementary.pdf]
